# Seagrass Herbivory Levels Sustain Site-Fidelity in a Remnant Dugong Population

**DOI:** 10.1371/journal.pone.0141224

**Published:** 2015-10-22

**Authors:** Elrika D’Souza, Vardhan Patankar, Rohan Arthur, Núria Marbà, Teresa Alcoverro

**Affiliations:** 1 Oceans and Coasts Program, Nature Conservation Foundation, Mysore, Karnataka, India; 2 Centre for Wildlife Studies, Bengaluru, Karnataka, India; 3 National Centre for Biological Sciences, Bangalore, Karnataka, India; 4 Department of Global Change Research, IMEDEA (CSIC-UIB), Institut Mediterrani d’Estudis Avançats, Illes Balears, Spain; 5 Centre d'Estudis Avançats de Blanes (CSIC), Blanes, Girona, Spain; Università della Calabria, ITALY

## Abstract

Herds of dugong, a largely tropical marine megaherbivore, are known to undertake long-distance movements, sequentially overgrazing seagrass meadows in their path. Given their drastic declines in many regions, it is unclear whether at lower densities, their grazing is less intense, reducing their need to travel between meadows. We studied the effect of the feeding behaviour of a small dugong population in the Andaman and Nicobar archipelago, India to understand how small isolated populations graze seagrasses. In the seven years of our observation, all recorded dugongs travelled either solitarily or in pairs, and their use of seagrasses was limited to 8 meadows, some of which were persistently grazed. These meadows were relatively large, contiguous and dominated by short-lived seagrasses species. Dugongs consumed approximately 15% of meadow primary production, but there was a large variation (3–40% of total meadow production) in consumption patterns between meadows. The impact of herbivory was relatively high, with shoot densities c. 50% higher inside herbivore exclosures than in areas exposed to repeated grazing. Our results indicate that dugongs in the study area repeatedly graze the same meadows probably because the proportion of primary production consumed reduces shoot density to levels that are still above values that can trigger meadow abandonment. This ability of seagrasses to cope perhaps explains the long-term site fidelity shown by individual dugongs in these meadows. The fact that seagrass meadows in the archipelago are able to support dugong foraging requirements allows us to clearly identify locations where this remnant population persists, and where urgent management efforts can be directed.

## Introduction

Large grazing mammals often form dense herds that migrate across vast stretches of landscape [1]. This grouping behaviour results in an uneven distribution of herbivores across the landscape, with a grazing pattern that continually shifts over time and space. In tropical seas, large herds of herbivorous dugongs graze over a seascape of hundreds of kilometres moving between meadows dominated by their preferred species [2,3]. Dugongs appear to select seagrass species that are high in nitrogen and low in fibre, characteristic of early successional plant species [4]. Meadows that sustain heavy dugong grazing are heavily altered in terms of plant productivity, community structure and nutrient content of the plant tissue [5,6]. One reason for this dramatic habitat alteration is that unlike most other herbivores (terrestrial or marine) that feed exclusively on leaves, dugongs usually feed on the entire plant, including shoots, rhizomes and roots. This is a particularly destructive form of feeding, resulting in meadows dominated by pioneering species maintained at low overall biomass by recurrent grazing [4]. At high foraging rates, this behaviour can result in a rapid depletion of the meadow biomass, triggering movements of dugongs once a meadow is overgrazed (with shoot density reductions up to 90%), with dugongs undertaking small- or large-scale migrations in search of more suitable forage grounds [2,7–11]. These movements are driven by a combination of factors including feeding preferences, seasonal and latitudinal temperature variations (which can also influence seagrass production), the quality and quantity of forage and meadow distribution within the larger landscape [1,12]. Once abandoned, seagrass meadows can recover their primary successional species relatively quickly, enabling dugongs to repeatedly return to the area, maintaining meadows with these preferred species, a behavior known as cultivation grazing [8]. Understanding the way dugongs use their habitat and move between foraging grounds has important consequences for the management of these large herbivore populations since they can result in populations widely distributed in space, and with often unpredictable distributions.

While these grazing and movement patterns have been clearly documented in high-density populations of dugongs, such as those found in Australia [11], these large herd sizes and population numbers are likely unique to this region. In other parts of the dugong's range (South-East Asia to the Red Sea), the species is undergoing dramatic extinctions and populations have been heavily decimated [13,14]. Among the many impacts of this reduction is that dugongs are now rarely seen in herds and are more typically sighted in small groups of one or a few individuals [9,15]. The few studies that have tracked movement patterns of individuals in these populations suggest that, rather than being fixed herds with strong social bonds, dugongs travel in small, loose feeding assemblages, regularly re-cropping restricted seagrass swards [9]. However, solitary individuals have also been observed to modify their behaviour in relation to predation risk by optimizing energy gain and safety by spending less time in more profitable but dangerous patches, or decreasing their use of risky feeding tactics that would increase net energy gain [16–18]. Understanding the habitat use of small groups or individual dugongs becomes critical as it can determine the movement behaviour or spatial persistence of this vulnerable species with important consequences for its conservation. Dugong-seagrass interactions could precipitate dugong movements or the seagrass ecosystem could shift in time. However, this will be highly dependent on the intensity and frequency of the herbivory as well as the ability of the meadow to cope with it (i.e. time window for recovery and seagrass re-colonisation rate).

In the Andaman and Nicobar archipelago, dugongs are one of the main herbivores in seagrass ecosystems. However, over the last 50 years, dugong populations have undergone drastic declines, and in some islands of the archipelago, they have been locally extirpated [19,20]. In the light of these decadal declines our main objective was to estimate present dugong habitat use, patterns of herbivory and the ability of seagrass meadows to cope with current herbivory pressure. We (i) tracked the size of dugong herds observed over the last 7 years, (ii) determined the characteristics of seagrass meadows that dugongs continue to use (44 meadows were surveyed), (iii) tracked the persistence of use (presence of feeding trails) in eight of these meadows over 4 years, (iv) measured the magnitude of dugong grazing through direct field measurements (herbivory versus primary production) in six of these meadows, and (v) used herbivory exclosures to determine the medium-term impact of dugong herbivory on seagrasses in three of these meadows.

## Methods

### Ethics statement

The dugong is listed under Schedule I of the Indian Wild Life Protection Act, 1972 which regulates all research on the species. We obtained appropriate research permits for our research from the Department of Environment and Forests, Port Blair. Since our research involved no animal handling or tissue sample collection no additional permits were required from the Ministry. Some of our sampling was conducted within Wildlife Sanctuaries, Protected Areas, National Parks and Tribal Reserves, and we obtained relevant entry permits from the Department of Environment and Forests and the Andaman and Nicobar Administration where appropriate.

Our work with local communities adhered to all standard scientific norms and our interview surveys were cleared by Nature Conservation Foundation's ethics committee. Our informants belonged to local settler and indigenous communities. Data collected from these surveys was limited to reporting dugong sightings in the area. No personal data was collected and all reported sightings were anonymised. Since literacy is low across all the communities we surveyed, the NCF Research Ethics committee waived the need for written informed consent from the participants, but we obtained verbal consents from each informant to record dugong presence. The only indigenous tribe we interacted with were the Nicobarese. In working with this tribe, we obtained the verbal consent of the Head of the Tribal Council of each village after explaining the objectives of the study and how the sighting records collected from the informants would be used.

### The study area

**Fig 1:**
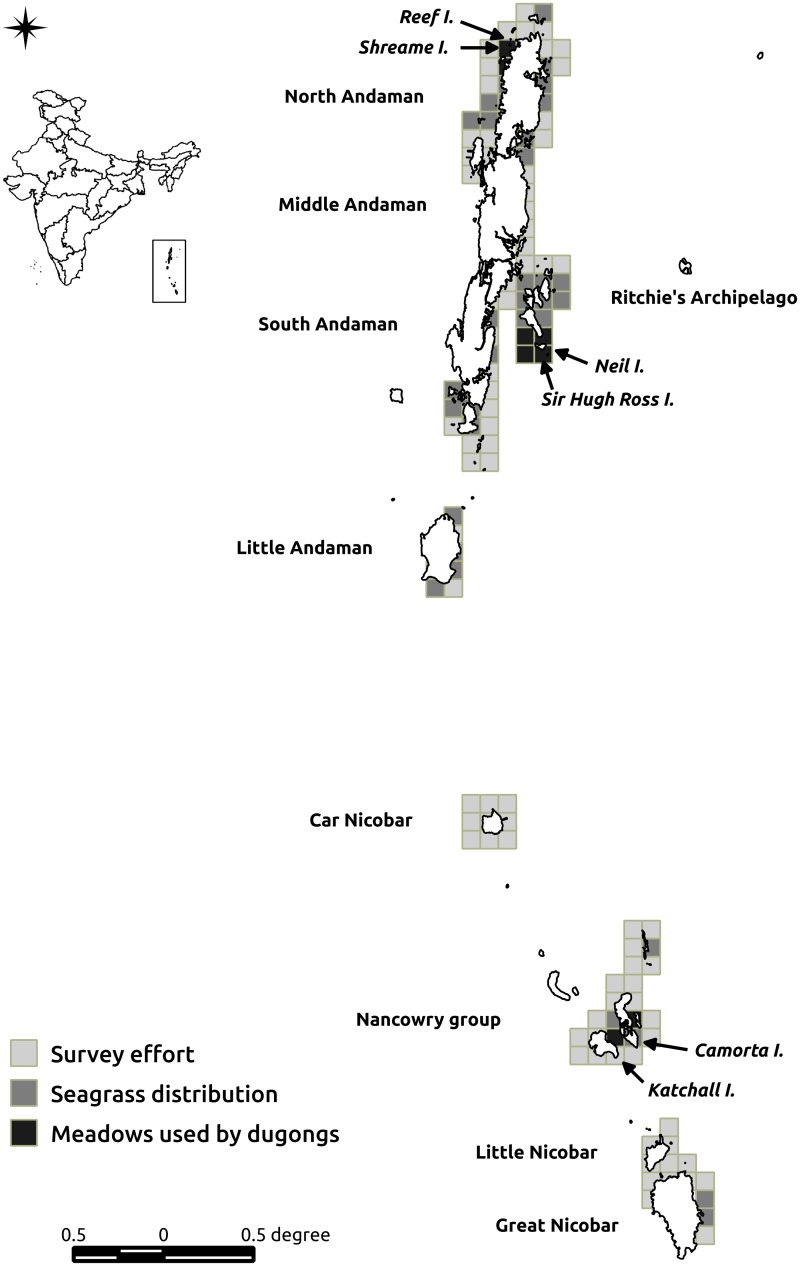
Map of the study area, the Andaman and Nicobar archipelago, India. The light grey cells indicate the survey effort, the dark grey, the present distribution of all seagrass meadows and the black show the meadows with confirmed dugong presence.

The Andaman and Nicobar archipelago is situated in the southeastern region of the Bay of Bengal between latitudes 6°45' N and 13°41' N and longitudes 92°12' E and 93°57' E. This archipelago comprises more than 350 islands, occupying an area of 8,249 km^2^ with a total coastline of 1,962 km that includes the Andaman group (>325 islands, 24 inhabited, 6,408 km^2^) and the Nicobar group (21 islands, 13 inhabited, 1,841 km^2^ [21], Fig 1). The climate is tropical and the region receives rainfall from the southwest and northeast monsoon winds. The islands have highly diverse terrestrial and marine ecosystems, comprising evergreen and littoral mangrove forests, extensive seagrass meadows, fringing coral reefs and active volcanic islands. The islands are inhabited by indigenous tribes of negrito (Onge, Jarawa, Great Andamanese, Sentinelese) and mongoloid (Nicobarese, Shompen) origins and recent (c. 80–100 years) immigrant settlers from mainland India, Bangladesh, Sri Lanka and Myanmar. Agriculture, livestock rearing, fisheries and plantation forestry are the main occupations in the islands, and the indigenous tribes still significantly depend on forest produce and hunting, including dugong hunting [22,23]. These islands were severely hit by the tsunami of 2004 that led to uplift of land in the northern islands by almost a meter and submergence of the southern islands by about 3 m [24]. This altered coastlines and resulted in the loss of several coastal ecosystems [25].

### Dugong population size and distribution: Informer networks and archipelago-wide survey

We maintained a comprehensive database of dugong sighting records from 2007–2013 to determine the location, numbers and herd size across the archipelago. Data on sightings were collected from an informer network that was established across the islands. Our network comprised of 19 key informants that included fishers, tourist boat operators and dive-centers. These informants covered the Nancowry group, North, Middle and South Andaman and Ritchie's archipelago (Fig 1). We attempted to validate every sighting with direct field visits, detailed follow-up interviews with informants and surveys of meadows to look for grazing signs. In addition, we conducted an archipelago-wide boat-based survey, covering c. 75% of the Andaman and Nicobar coastline. Given the length of the coastline, the briefness of the fair season and the logistic difficulties of surveying these islands, this survey was conducted over two years (2010 and 2011), co-incident with the period of highest seagrass productivity (i.e., summer) to increase our chances of sighting dugongs that grazed in meadows. While this was designed to survey the distribution and characteristics of seagrass meadows across the archipelago (see later), we also used these surveys to document the presence of dugongs through direct visual sightings [20]. Wherever possible, sightings were complemented with photographs to verify the presence of dugongs at a particular location. In addition, we used characteristic flipper and tail-fluke markings to maintain a database of individual dugong identifications and avoid re-counting individuals. For every sighting, data on number of individuals, individual size, sex (wherever possible), location of nearby foraging grounds, and behavioural state was collected.

### Distribution and characteristics of seagrass meadows used as foraging grounds: Archipelago-wide survey

During the archipelago-wide survey (conducted in 2010 and 2011, see earlier) we located and sampled seagrass meadows, recorded dugong use and determined characteristics of each meadow (Fig 1). Primary data on meadow presence was collected with boat-based surveys, using a systematic spatial sampling approach. All sites with sandy or muddy substrates and gradual slopes (likely habitats for seagrass) were sampled. Reefs and rocky areas were not surveyed since the probability of meadows occupying these substrates is low. In addition, we did not survey potential seagrass meadows adjacent to dense mangrove areas, although with possible suitable substrate, due to the likelihood of encountering crocodiles, which are abundant in the archipelago. All suitable habitats were first checked for the presence of seagrass meadows with rapid visual in-water surveys. When a meadow was located, we recorded its depth, extent, exposure to wave action, composition and density, level of patchiness and use by dugongs. In order to ensure that our archipelago-wide survey was as comprehensive as possible, we also used prior information on the distribution of seagrass meadows available in the literature [26,27] as well as the knowledge of local fishermen and regular sea-farers to locate and sample seagrass meadows present at every location.

#### Depth, extent and wave exposure

Using a hand-held depth finder, we measured the depth of a meadow at four random locations within the area. We classified meadows into three depth categories, 1–5 m, 5–10 m and 10–15 m. Free swims were conducted to locate the periphery of the meadow and the two main axes: length and width were measured to determine the approximate areal extent. Based on it's exposure to wave action, we classified meadows into three categories, exposed, partially exposed and sheltered.

#### Composition and density

We estimated seagrass species composition and shoot densities in each meadow in three random quadrats of 20 × 20 cm^2^ collected by SCUBA diving. Meadow composition was classified as 1: *Halophila* sp. (*Halophila ovalis* and *Halophila minor*) dominated (95% abundance) or *Halodule* sp. (*Halodule uninervis* and *Halodule pinifolia*) dominated (95% abundance), 2: *Halophila* sp. + *Halodule* sp. co-dominants (either species <95% abundance), 3: *Halophila* sp. and/or *Halodule* sp. plus other species (*Thalassia hemprichii*, *Cymodocea rotundata*, *Cymodocea serrulata*, *Enhalus accoroides*, *Syringodium isoetifolium*); and 4: mixed meadows without *Halophila* sp. and *Halodule* sp.

#### Meadow patchiness and level of dugong use

We assessed meadow patchiness using four 50 m strip transects with scuba diving along which we recorded transitions in benthic cover (seagrass to sand/others) at a minimum interval ≥20 cm. We estimated the percent seagrass cover and classified meadows with patchy seagrass cover (<50%) as fragmented, and contiguous meadows with relatively uniform cover (>50%). The transects were always deployed inside the seagrass meadows, and were truncated if the edge of the meadow was encountered. Each meadow was also surveyed for dugong feeding signs along these transects (presence of dugong feeding trail or non-presence). To avoid misidentification of feeding signs, before the survey, we familiarized ourselves with characteristics of typical feeding trails (c. 20 cm wide) by following feeding dugongs and examining the signs they left in their wake. We used data on habitat covariates (detailed above) to determine factors that influenced habitat use.

### Determining dugong presence and the persistence of habitat use

In order to assess seasonal and inter-annual variability of dugong presence we established an informer network across the eight meadows where dugong feeding signs were observed during our extensive surveys (Katchall, Camorta, Neil 1, 2 and 3, Sir Hugh Ross, Radhanagar, Reef, Fig 1). Links were built with local fishers, boat owners, dive operators, other researchers and local community members who frequented these coasts and whose information could be relied on. A minimum of four informants covered each of these sites and each one was contacted every month to inquire about sightings of dugongs. Data was collected for four years from 2010 to 2013. Each year has three seasons, winter (October–January), summer (February–May) and monsoon (June–September).

Meadow use was confirmed based on the presence of feeding trails (using transect methods described above) or direct sightings, and was repeated at intervals of four months each year to avoid the influence of seasonal variations in habitat use. We spent a week in each meadow. Meadows with direct sightings or feeding trails on at least two occasions within a season were recorded as ‘presence’ for that season and on two or three seasons within a year were recorded as a ‘presence’ for that year. In some periods (monsoon) data could be not obtained due to rough weather conditions prevailing during the monsoons or the remoteness of some sites (absent data).

### Primary production and dugong herbivory in seagrass meadows

#### Measuring primary production

To measure primary production we selected three meadows (Neil 1, 2, 3) dominated by *Halophila ovalis* and which could be accessed throughout the year. At each meadow we randomly marked thirty apical shoots of *Halophila ovalis* with cable ties. The shoots were retrieved after 5 days and the increase in rhizome length and the number of new shoots produced was measured. This procedure was repeated in all three meadows in 2010 December, March and June. The number of apical shoots in a given area was also counted within four 20 × 20 cm^2^ randomly placed quadrats at 6 meadows (Katchall, Camorta, Neil 1, 2, 3, Reef) in which dugong herbivory was also later estimated. Growth rate was calculated as the average number of shoots produced by an apical rhizome per day and in a season meadow and this value was later multiplied by the number of apical shoots per unit area (1m^2^) in each of the four quadrats to obtain the rate of production of new shoots per unit area (shoots m^-2^ d^-1^) for each sampling period and meadow [28]. We tested for differences in primary production between meadows using a one-way ANOVA with site as a fixed factor. In addition, we tested for differences in primary production between sampling times using a one-way ANOVA with sampling time (December, March, June) as a fixed factor and site as a random factor (n = 6).

#### Measuring dugong herbivory

Dugong herbivory was measured in a subset of seagrass meadows (Katchall, Camorta, Neil 1, 2, 3, Reef), all dominated by *Halophila ovalis* where feeding signs were observed (n = 6 meadows). We chose *H*. *ovalis* meadows because 7 of the 8 meadows where feeding signs were observed were dominated by this species. Dugong herbivory (shoots m^-2^ d^-1^) was estimated by measuring (i) area occupied by the feeding trails present in each meadow within a unit area, (ii) biomass removed by dugongs in the feeding trails and (iii) the duration of feeding trails (i.e. time window within which a feeding trail can be visually recognized). Herbivory was measured between February and May, 2010.

The area of the meadow covered by feeding trails was measured in six 1 × 1 m^2^ random quadrats in meadows used by dugongs. We then selected four feeding trails per meadow and in each feeding trail we measured shoot densities within a 20 × 20 cm^2^ quadrat established inside (4 replicates) and outside (4 replicates), adjacent to the feeding trail. The seagrass biomass consumed by dugongs in the feeding trails was estimated as the difference in shoot density inside and outside the feeding trails. We estimated the duration of a feeding trail by marking 3 fresh feeding trails (characterized by a depression caused by grazing action and with fresh sediment still on the shoots) in three accessible seagrass meadows that were used by dugongs (dominated by *Halophila ovalis*, i.e. Neil 1, 2, 3). We re-visited these selected feeding trails at a 2-day interval until the entire trail was covered with new shoots and could not be distinguished visually from the rest of the meadow. The estimated feeding trail duration was 8.5 ± 0.33 days (c. 9 days, n = 9 feeding trails in 3 different sites) on average. We did not observe re-grazing on a feeding trail during the estimated feeding trail duration.

Finally, the rate of dugong herbivory was measured for each meadow using the following formula: Rate of herbivory (shoots m^-2^ d^-1^) = [(S_0_—S_1_) × A_f_ / t_f_], where, S_0_ = shoot density outside feeding trails, S_1_ = shoot density within feeding trails, A_f_ = area of feeding trail per total meadow area, t_f_ = duration of feeding trail.

This measure of herbivory is a conservative estimate of actual herbivory rates since it does not account for older feeding trails that could have been difficult to detect. We used this method as a robust conservative measure that could be employed rapidly for large-scale surveys.

Finally we compared the rate of herbivory obtained at each feeding site with the corresponding rate of shoot production for that location during the same sampling period to minimize seasonal influences on our comparisons. We compared rates of herbivory between meadows using a one-way ANOVA.

#### Effect of dugong herbivory on seagrasses

To test the effect of dugong herbivory on seagrass ecosystems we conducted an herbivory-exclosure experiment in three seagrass meadows dominated by *Halophila ovalis*. This experiment was done over a period of four months (March-June, 2011). We selected three seagrass meadows located around Neil Island, that were easily accessible and consistently used by dugongs during the period of the study. We created four 1 × 1 m^2^ dugong foraging exclosures at each meadow using a mesh of fishing lines (with a very wide mesh size to avoid light reduction while restricting dugong access[29]) and PVC pipes. At the start of the experiment (time T_0_), shoots density was measured within 20 × 20 cm^2^ quadrats inside (control), in the center of the exclosure, and outside (treatment) the exclosures. We restricted our sampling to the centre of the exclosure to avoid edge effects that would have required additional procedural controls and been difficult to establish in these meadows; this protocol can only be employed when significant shading is not an issue [30]. Four months later, the exclosures were revisited and shoot density inside and outside the exclosures was measured again. Controls and treatments were compared using a paired T-test at time T_0_ and T_1_ to determine the impact of dugong herbivory on seagrasses in the three meadows.

## Results

### Population size and distribution

We recorded a total of 15 dugongs across the archipelago (Table 1) over a period of seven years. At these locations we identified 5 individuals from fin and fluke markings from which we had multiple direct sightings of the same individuals frequenting the same location. The maximum herd size observed had 2 dugong individuals (Table 1).

**Table 1:** Number of dugongs sighted over a period of seven years across the Andaman and Nicobar archipelago.

Island name	Number of dugongs sighted							Total number of individually identified dugongs sighted
	2007	2008	2009	2010	2011	2012	2013	
Reef island	0	0	0	0	0	2	0	2 unknown
Havelock (Radhanagar)	1	1	0	0	0	0	0	1
Neil	2	2	2	2	2	2	2	2
Sir Hugh Ross	1	1	1	1	1	1	1	1
Inglis	0	0	0	0	0	2	0	2 unknown
South Andaman (Kodiaghat/ Burmanullah)	0	1	1	1	0	0	0	1
South Andaman (Wandoor)	0	0	0	0	0	2	0	2 unknown
Trinket	0	0	0	2	0	0	0	2 unknown
Nancowry	0	0	0	1	0	0	0	1 unknown
Teressa	0	0	1	0	0	0	0	1 unknown

The last column corresponds to the total number of observed individuals identified using flipper and tail-fluke markings. Animals sighted but not individually identified were marked as 'unknown'.

### Distribution and characteristics of seagrass meadows used as foraging grounds

We surveyed 44 seagrass meadows across the Andaman and Nicobar archipelago (Fig 1), occupying a total area of 0.712 km^2^, which were either mono-specific or multi-specific in seagrass composition (Table 2). We recorded a total of nine seagrass species: *Enhalus acroides*, *Syringodium isoetifolium*, *Thalassia hemprichii*, *Halophila ovalis*, *Halophila minor*, *Halodule uninervis*, *Halodule pinifolia*, *Cymodocea rotundata* and *Cymodocea serrulata*. Of the meadows surveyed, eight (c. 18.18% of surveyed meadows) were used by dugongs, evident from the distinct feeding trails observed at these sites. These meadows were located around Reef (1 meadow), Shreame (1 meadow), Neil (3 meadows), Sir Hugh Ross (1 meadow), Camorta (1 meadow) and Katchall (1 meadow) islands (Fig 1). All eight meadows were monospecific, seven of them composed of *Halophila ovalis*, and one of *Halodule pinifolia*. These meadows were relatively large in size and had a continuous cover of seagrass shoots. The level of fragmentation, size of the meadow and species composition appeared to be the most important factors determining habitat use (Table 2).

**Table 2:** The location and characteristics of all seagrass meadows surveyed.

Island	Area (m²)	Depth (m)	Wave exposure	shoot density (shoots m^-2^)	Species composition	Meadow patchiness	Dugong presence
Great Nicobar (B-Quarry)	1500	5–10	exposed	1256.25±167.82	*Halodule pinifolia*	fragmented	0
Camorta (Derring Bay)	60000	0–5	sheltered	3333.33±1794.74	*Halodule pinifolia*	fragmented	0
Camorta (Kardip)	7500	0–5	exposed	2875±828.77	*Halophila ovalis*, *Halodule uninervis*, *Enhalus acoroides*	fragmented	0
Camorta (Aalinchi)	1250	0–5	sheltered	3331.25±390.16	*Halophila ovalis*, *Halophila minor*	fragmented	0
**Camorta (Bada Enaka)**	**5000**	**0–5**	**partially exposed**	**1333.33±164.15**	***Halophila ovalis*, *Halodule uninervis***	**contiguous**	**1**
Nancowry (Champian)	100	0–5	sheltered		*Enhalus acoroides*	fragmented	0
Nancowry (Hitui)	3750	0–5	sheltered	2975±648.32	*Enhalus acoroides*, *Halodule uninervis*, *Halophila ovalis*	fragmented	0
Nancowry (Malacca)	1250	0–5	partially exposed	5081.25±835.44	*Halodule uninervis*, *Halophila ovalis*, *Enhalus acoroides*, *Halodule pinifolia*	fragmented	0
Nancowry (Altaiyak)	15000	0–5	exposed	4443.75±867.37	*Cymodocea rotundata*, *Halophila ovalis*, *Syringodium isoetifolium*, *Cymodocea serrulata*, *Thalassia hemprichii*, *Enhalus acoroides*, *Halodule uninervis*	fragmented	0
**Katchall**	**15000**	**0–5**	**partially exposed**	**2306.25±554.09**	***Halodule uninervis*, *Halophila ovalis***	**contiguous**	**1**
Tillangchong (East Police Camp)	750	0–5	partially exposed	2418.75±289.46	*Halodule pinifolia*, *Halophila ovalis*	fragmented	0
Tillangchong (Floatsam)	5000	5–10	sheltered	1737.5±139.38	*Halodule pinifolia*	contiguous	0
Trinket	250	5–10	exposed	-	*Halophila minor*	fragmented	0
Little Andaman (Butlet Bay)	5000	10–15	exposed	1781.25±164.37	*Halophila minor*	fragmented	0
Little Andaman		0–5	exposed	-	*Cymodocea rotundata*	fragmented	0
Little Andaman (South Bay)	5000	0–5	exposed	-	*Halophila ovalis*	fragmented	0
MGMNP	patches	0–5	partially exposed	-	*Halophila ovalis*	fragmented	0
Rutland	5000	0–5	sheltered	-	*Halophila ovalis*	fragmented	0
Tarmugli (north-east)	2500	0–5	partially exposed	-	*Enhalus acoroides*, *Halodule uninervis*, *Halophila ovalis*	fragmented	0
Tarmugli (south-east)	3750	0–5	partially exposed	-	*Enhalus acoroides*, *Halodule uninervis*, *Halophila ovalis*	fragmented	0
South Andaman (Haddo Foreshore Road)	2500	0–5	partially exposed	1943.75±269.52	*Halodule uninervis*, *Halophila ovalis*, *Thalassia hemprichii*	fragmented	0
South Andaman (North Wandoor)	5000	0–5	partially exposed	-	*Halophila ovalis*	fragmented	0
South Andaman (Kanaidera)	2500	0–5	exposed	-	*Halodule pinifolia*	fragmented	0
South Andaman (Mahuadera)	2500	0–5	exposed	-	*Halodule pinnifolia*	fragmented	0
**Neil 1**	**40000**	**10–15**	**exposed**	**2893.75±271.26**	***Halophila ovalis***	**contiguous**	**1**
**Neil 2**	**45000**	**10–15**	**exposed**	**3781.25±264.65**	***Halophila ovalis***	**contiguous**	**1**
**Neil 3**	**15000**	**10–15**	**exposed**	**1775±290.29**	***Halophila ovalis***	**contiguous**	**1**
**Sir Hugh Ross**	**20000**	**10–15**	**exposed**	**2568.75±168.13**	***Halophila ovalis***	**contiguous**	**1**
Havelock (Radhanagar)	60000	5–10	exposed	856.25±240.74	*Halophila ovalis*, *Halodule pinifolia*	fragmented	1
Henry Lawrence	30000	0–5	exposed	1150±200.78	*Halophila ovalis*, *Syringodium isoetifolium*, *Halodule pinifolia*, *Thalassia hemprichii*, *Halodule uninervis*, *Enhalus acoroides*	fragmented	0
Middle Andaman (Below Shoal bay)	5000	0–5	exposed	-	*Halodule pinifolia*	fragmented	0
Strait island	2500	0–5	partially exposed	-	*Halodule pinifolia*	fragmented	0
Mayabunder (Bay)	250000	0–5	sheltered	3781.25±1006.61	*Halodule pinifolia*	fragmented	0
Mayabunder (Pokkadera)		0–5	partially exposed	-	*Halodule pinifolia*, *Halodule uninervis*, *Halophila ovalis*, *Halophila minor*, *Cymodocea rotundata*, *Thalassia hemprichi*	fragmented	
North Reef (East)	500	0–5	partially exposed	-	*Halophila ovalis*	fragmented	0
Latuche (East)	500	0–5	exposed	-	*Halophila ovalis*	fragmented	0
Craggy	2500	5–10	partially exposed	593.75±76.63	*Halodule pinifolia*	fragmented	0
Diglipur (Kalipur)	2500	0–5	exposed	-	*Thalassia hemprichii*, *Cymodocea rotundata*	fragmented	0
Ross Smith	12500	0–5	partially exposed	1225±139.19	*Halodule pinifolia*, *Halophila ovalis*	fragmented	0
Temple Island	625	0–5	exposed	641.66±83.33	*Halophila ovalis*, *Halodule pinifolia*	fragmented	0
North Andaman (Casuarina Bay)	3750	0–5	partially exposed	2337.5±188.33	*Halophila minor*, *Halophila decipiens*	fragmented	0
**Shreame**	**5000**	**0–5**	**partially exposed**	**3350±119.46**	***Halodule pinifolia***	**contiguous**	**1**
**Reef Island**	**1250**	**0–5**	**exposed**	**1531.25±129.25**	***Halophila ovalis***	**contiguous**	**1**
East Island	500	0–5	exposed	-	*Halophila ovalis*	fragmented	0

The rows highlighted in boldface are meadows with confirmed dugong usage.

### Dugong presence and the persistence of habitat use

Informants reported sightings of dugongs in and around some of the monitored meadows almost throughout the year (Table 3) during all seasons. There were a few gaps in records during the monsoons as not many informants ventured into the sea and sightings from the shore tended to be difficult due to rough seas prevalent at that time of the year.

**Table 3:** Seasonal and inter-annual use of seagrass meadows by dugongs.

Site	Source	Seasonal and annual presence of dugong															
		2010				2011				2012				2013			
		W	S	M	Presence for 2010	W	S	M	Presence for 2011	W	S	M	Presence for 2012	W	S	M	Presence for 2013
Camorta	Informer network	A	A	A	A	A	A	A	P	A	A	A	-	A	A	A	-
	Field surveys	A	A	-		P	P	-		-	-	-		-	-	-	
Katchall	Informer network	A	A	A	P	A	A	A	A	A	A	A	-	A	A	A	-
	Field surveys	P	P	-		A	A	-		-	-	-		-	-	-	
Neil 1	Informer network	P	P	P	P	P	P	P	P	P	P	P	P	P	P	P	P
	Field surveys	P	P	A		P	P	A		P	P	-		P	P	-	
Neil 2	Informer network	P	P	P	P	P	P	P	P	P	P	P	P	P	P	P	P
	Field surveys	P	P	A		P	P	-		P	P	-		P	P	-	
Neil 3	Informer network	P	P	P	P	P	P	P	P	P	P	P	P	P	P	P	P
	Field surveys	P	P	A		P	P	A		P	P	-		P	P	-	
Sir Hugh Ross	Informer network	P	P	P	P	P	P	P	P	P	P	P	P	P	P	P	P
	Field surveys	P	P	-		P	P	-		P	P	-		P	P	-	
Shreame	Informer network	A	A	A	-	A	A	A	-	A	A	A	-	A	A	A	-
	Field surveys	-	-	-		-	P	-		-	-	-		-	-	-	
Reef	Informer network	A	A	A	-	A	A	A	-	A	A	A	-	A	A	A	-
	Field surveys	-	-	-		-	P	-		P	-	-		-	-	-	

(W = Winter, S = Summer, M = Monsoon, P = Present, A = Absent, '-' = Data unavailable). Dugong presence from the informer network was based on monthly data. Field surveys were conducted once every four months.

We found signs of dugongs feeding in almost 50% of the meadows (meadows located around Neil and Sir Hugh Ross Islands) throughout the four-year period during which we monitored the meadows (Table 3). In some meadows (Katchall and Bada Enaka) dugong signs were observed only in a single year during the four-year period. However, whether these meadows ceased or continued to be used by dugongs could not be investigated further due to the remoteness of the site affecting accessibility. Re-use of sites by the same animals has to be checked for some sites as animals were not sighted and it was difficult to confirm this based on feeding trails.

### Primary production

**Fig 2:**
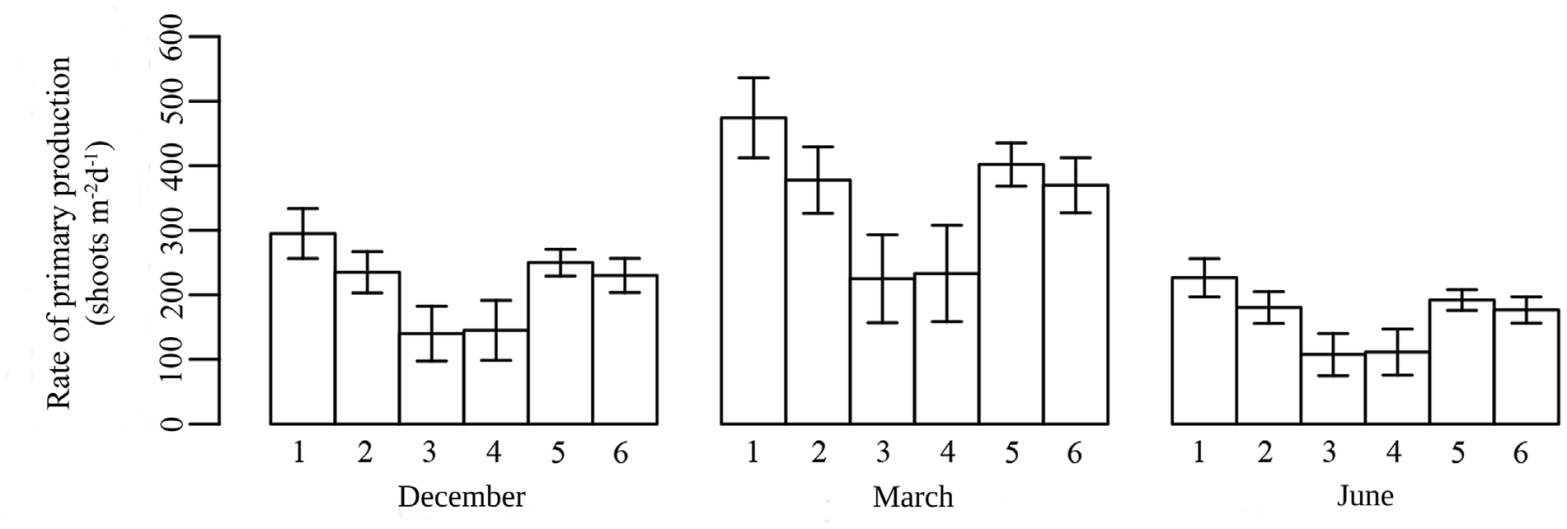
Rate of primary production across all meadows measured in the three sampling periods (December, March and June). The numbers on the X-axis correspond to the meadows where measurements were made (1 = Katchall, 2 = Neil1, 3 = Neil2, 4 = Neil3, 5 = Camorta, 6 = Reef). Error bars are standard errors.

There was clear variation in the growth rate of *Halophila ovalis* species between sampling months, with March being the period of highest growth, dropping to its lowest during June (Table 4). The growth rate observed during December, was 0.8 ± 0.4 shoots rhizome-apex^-1^ d^-1^ (n = 5), while in March it was 1.28 ± 0.47 shoots rhizome-apex^-1^ d^-1^ (n = 60) which declined considerably during June to 0.61 ± 0.23 shoots rhizome-apex^-1^ d^-1^ (n = 28). In addition, there were considerable site-level differences in this pattern (Table 5). Overall, primary production was highest at Katchall and lowest at Neil (site 2) (Fig 2 and Table 5).

**Table 4:** Differences in seagrass primary production between sampling times (March, December and June).

	Factor	df	SS	MS	F ratio	p value
Sampling time	Month	2	419969	209985	**20.87**	<0.001**
	Error	69	694236	10061		

The table shows the results of a one-way ANOVA with site as a random factor (n = 6). Df = degrees of freedom, SS = Sum of Squares, MS = Mean sum of Squares. Significant p values (p<0.001) marked in boldface and asterisks (**). Tukey HSD: March> December = June

**Table 5:** Differences in herbivory rates and seagrass primary production between sampling sites.

	Factor	df	SS	MS	F ratio	p value
Herbivory	Site	5	15216	3043	**6.97**	<0.001**
	Error	18	7855	436		
Primary production	Site	5	285082	57016.4	**4.54**	<0.001**
	Error	88	829123	12562		

The table shows the results of one-way ANOVAs with site as a fixed factor (n = 6). Df = degrees of freedom, SS = Sum of Squares, MS = Mean sum of Squares. Significant p values (p<0.001) marked in boldface and asterisks (**). Tukey HSD (herbivory): Neil1> rest of sites. Tukey HSD (production): Katchall ≥ Camorta, Neil1, Reef ≥Neil3, Neil2.

### Magnitude of dugong herbivory

**Fig 3:**
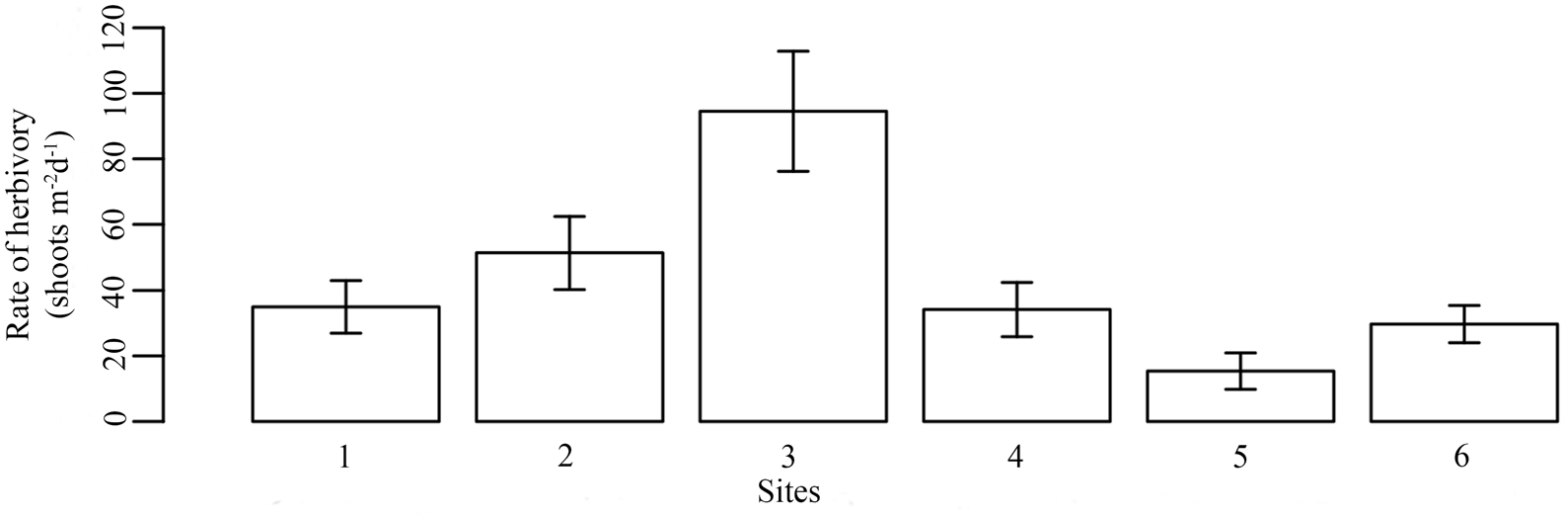
Rate of dugong herbivory measured across six seagrass meadows between February and May. The numbers on the X-axis correspond to the meadows where measurements were made (1 = Katchall, 2 = Neil1, 3 = Neil2, 4 = Neil3, 5 = Camorta, 6 = Reef). Error bars are standard errors.

**Fig 4:**
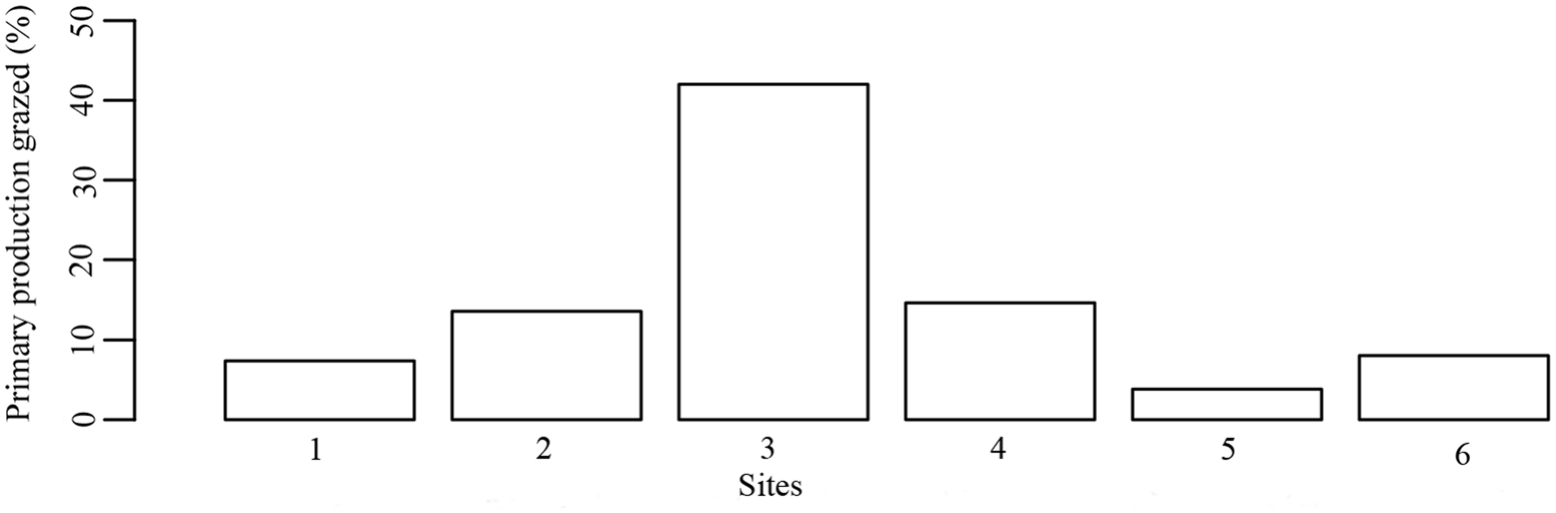
Percentage of primary production grazed by dugongs in six meadows. This was measured in the period of highest productivity. The numbers on the X-axis correspond to the meadows where measurements were made (1 = Katchall, 2 = Neil1, 3 = Neil2, 4 = Neil3, 5 = Camorta, 6 = Reef).

Dugong feeding trails occupied an area ranging from 0.20 m^2^ to 0.33 m^2^ of the area of the random quadrats on the six studied sites. Within dugong feeding trails, shoot densities were on average 789.58 ± 109.36 shoots m^-2^ compared to 2061.46 ± 208.86 shoots m^-2^ (n = 24 feeding trails in 6 different sites) outside the feeding trails, representing an average reduction of 58.76% in abundance. Our estimates of herbivory indicate that dugong off-take varied considerably between meadows accounting for between 15.37 ± 5.54 to 94.51 ± 18.32 shoots m^-2^ d^-1^ at the meadow level (mean ± S.E., Fig 3 and Table 5). The highest herbivory was observed at Neil 2 and the lowest at Camorta. The rate of herbivory averaged 14.91 ± 5.66% of primary production but varied widely between sites, ranging from 3.82% to 42% of primary production (Fig 4).

### Effect of dugong herbivory on seagrasses

**Fig 5:**
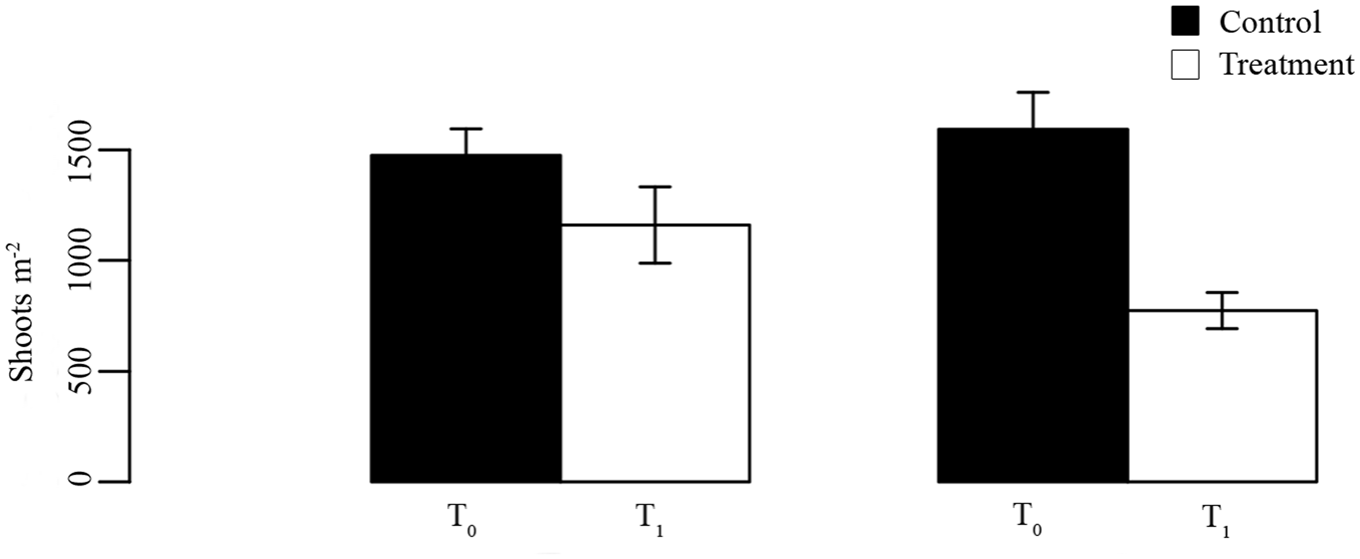
Effect of dugong herbivory on the shoot density of *Halophila ovalis*. Shoot densities were measured inside and outside experimental exclosures at the start of the experiment (T0) and 4 months later (T1). Error bars are standard errors.

At the onset of the experiment, there was no significant difference between shoot abundance inside and outside the exclosures (p = 0.20, t = 1.36). After 110 days, average shoot densities in the dugong exclosure plots increased by 11.86% between sampling intervals, from 1475 ± 120.14 to 1592.86 ± 167.32 shoots m^-2^ d^-1^ (n = 6). In contrast, in plots exposed to dugong grazing, average shoot densities showed a notable decrease of 33.18% from 1160 ± 172.77 to 775 ± 81.77 shoots m^-2^ d^-1^ (n = 6, p = 0.002, t = 4.96 Fig 5).

## Discussion

In seven years of almost continuous observation, there were very few independent direct sightings of dugongs across the Andaman and Nicobar archipelago, all limited to solitary individuals or pairs. They appeared to reuse a few meadows persistently over the 4 years of our observation; these meadows were characteristically large, contiguous, mostly exposed and dominated by short-lived seagrass species. This site fidelity meant that dugongs could consume up to 40% of primary production in some used meadows, resulting in a near halving of shoot densities with repeated grazing. The ability of the meadow to cope with this level of repeated off-take at low densities of dugongs is probably critical to support persistent foraging. At the same time, this high spatial fidelity enables a clear identification of foraging areas for conservation that may be vital in managing this remnant population.

The dugong population in the Andaman and Nicobar archipelago appears to be largely restricted to solitary individuals with a few pairs that persistently graze in isolated pockets across the island chain. Solitary grazing males and females dominated our sightings, and mother and calf sightings were rare. This contrasts with social systems observed in regions with relatively high dugongs densities (such as in Australia) which are characterized by large, mostly female foraging herds accompanied by young individuals of mixed sex; adult males at these locations tend to be solitary [31]. This herd-based social system may be driven by predation pressure, which is diluted in aggregations. However, travelling in groups could also facilitate a more efficient use of resources by migrant species searching for high density, nutrient rich forage [32,33]. Interviews with older fishers and indigenous communities in the Andaman and Nicobar archipelago indicate that larger dugong herds were relatively common in the past (herd sizes of 5–7 individuals c. 30 years ago). Our observations indicate that this herd-based social system may have broken down in the recent years, coinciding with an important loss in occupancy across the archipelago [20].

Though they may differ in their social systems from more pristine populations, dugongs in the Andaman and Nicobar archipelago make foraging choices very similar to animals in other regions, drawn to meadows dominated by pioneering seagrass species, *Halophila ovalis* and *Halodule uninervis [34*], often chosen by dugongs for their low-fibre and high-nitrogen composition [8,35] as observed in Indonesia [3], Thailand [34] and Australia [4]. Our results cannot determine if meadow composition is itself driven by grazing facilitation by dugongs or if the animal restricts its choice to meadows already dominated by pioneers. Dugongs are known to graze down entire meadows, abandoning them for a while, returning once the grazed site has recovered. This type of “cultivation grazing” is capable of causing a disturbance so intense, it precipitates compositional shifts towards fast-growing species, retarding the growth of less-preferred and slower-growing species [34]. Preen [4] argues that this profound modification can only occur when animals graze in large herds. However, our results show that even at low numbers, dugong herbivory may still be capable of modifying seagrass meadows with sustained grazing activity, maintaining them with species preferred by dugongs.

While meadow species composition is critical to habitat choice, our results indicate that dugongs additionally show a clear preference for contiguous meadows over fragmented ones and exposed over sheltered meadows. The Andaman and Nicobar islands have a high sediment regime that influences meadow dynamics resulting in a matrix of small meadow patches (personal observations). These patchy seascapes appear to be clearly avoided by dugongs, perhaps because they are not very effective for the characteristic foraging that dugongs employ, grazing continuously across long uninterrupted stretches of meadow [4]. Dugongs apparently selectively choose contiguous meadows dominated by fast growing species, with high nutrients, where they can actively forage to meet daily dietary requirements. There is evidence that herbivores increase foraging efficiency by choosing pastures of high density and nutrient rich forage as foraging velocity decreases and intake rate increases in these areas [32,33]. In the Andaman and Nicobar, most of the meadows used by dugongs were either partially or completely exposed. In the high sediment regimes of the archipelago, it is likely that sediment loads play an important role in determining the distribution of meadows, as well as their species composition. We are unable to determine how exposure, sediment and species composition interact to influence dugong use of meadows, but this is clearly an area that warrants further investigation.

Our study indicates that dugongs show a high amount of fidelity to foraging grounds even when several other apparently suitable meadows were present in the area. Only 20% of surveyed meadows were used and while only a subset of these were suitable in terms of size, species composition and degree of fragmentation, the dugong population in these islands appear more constrained by population size than by food resource. This site fidelity has also been observed in other areas with small populations of dugong[36]. Without tracking individuals, we cannot assert that the same individuals were responsible for the feeding trails observed over time at each meadow, but in the few instances where we have been able to spend several months observing the same meadow (Neil 1 and Neil 2), we recorded two individually-identified dugongs repeatedly feeding at these meadows. Critical to this fidelity is the persistence of the habitat itself, and its ability to cope with sustained grazing activity. The rate of herbivory, measured with our herbivory estimates, indicates that dugong herbivory represented an average of 15% off-take of seagrass primary production. While it can be argued that our herbivory measures may be a conservative underestimate of actual rates of herbivory, it is clear that seagrass meadows can potentially cope with significantly higher rates of off-take. Seagrass consumption observed in areas where herbivory (by fish and green turtles, for instance) is very high were significantly greater than our estimates[37,38]. In the case of the dugong however, it is not merely the rate of herbivory that matters but the highly destructive feeding mode that dugongs employ, uprooting the entire plant along their feeding trails. This highly destructive form of grazing can significantly reduce the meadow’s ability to recover; results from our herbivore exclosures indicate that even with the fairly moderate levels of herbivory experienced by these meadows, the effect of sustained grazing can reduce shoot densities by 50%. It must be stressed however that early successional species may be able to recover quickly from even these major reductions. Grazed shoot densities were well below patch abandonment levels observed in Australian meadows, where large dugong herds left meadows only after reducing shoot density to between 60–95% [4]. Meadows in the Andaman and Nicobar are potentially able to deal with the levels of herbivory they are subject to, promoting high local persistence by dugongs. In fact, where we have been able to follow a single individually-identified dugong for a long period, the animal returned almost daily to the same meadow to feed for 18 consecutive months.

The apparently predictable site fidelity we documented has important implications for management as it clearly identifies spatially explicit areas for conservation linked to meadows that dugongs use frequently. However, this conclusion must be circumscribed by a few important concerns. Critically, we have not measured dugong movements, nor can we completely confirm (apart for a few individuals) that the same dugongs are responsible for the observed persistence in habitat use. It is also clear that foraging decisions are only one in a suite of other factors that could drive dugong movements including male and female mating strategies, escape from predation, calf protection, anthropogenic noise, oceanography, among others. It is also unclear, in the absence of reliable estimates of current dugong populations, how much this putative reduced movement is associated with, and may even contribute to Allee effects, which may be critical to address if this population has to be rescued from local extinction. Understanding movement patterns by dugongs will require remote-tracking individuals, currently inconceivable under the strict environmental laws that protect dugongs in India. However, even if dugongs do undertake long-distance travel for feeding grounds or social interactions as observed elsewhere, the importance of protecting current foraging grounds is vital if this population has to be given the best chance to recover. Management efforts need to ensure that these identified meadows are given the strongest protection possible, curtailing boat traffic, net fishing and other activities that could directly impact dugongs. It will be additionally important to maintain and monitor meadow condition to ensure that critical forage resources are not degraded. While these measures will only provide protection within a potentially small part of its realized range, it is an urgent first step for this population.

Our study helps identify habitats key to the survival of dugongs in the Andaman and Nicobar Islands, while contributing to a wider understanding of the role large herbivores can play in marine systems. It highlights that even at low densities, mega-herbivores are still capable of engineering an ecosystem to maximise energetic gains. The complex interaction between these herbivores and the plants they consume can have important implications for foraging behaviour and movement patterns that may change dynamically as populations decline. Understanding these changes is critical while designing management interventions for remnant populations threatened with local extinction.
